# Children with autism spectrum disorder who demonstrate normal language scores use a bottom‐up semantic processing strategy: Evidence from N400 recordings

**DOI:** 10.1002/brb3.3158

**Published:** 2023-07-21

**Authors:** Lee Phan, Alina Tariq, Garbo Lam, Maaz Mirza, Dylan Paiva, Milan Lazic, Zahra Emami, Evdokia Anagnostou, Karen A. Gordon, Elizabeth W. Pang

**Affiliations:** ^1^ SickKids Research Institute Toronto Ontario Canada; ^2^ University of Toronto Department of Paediatrics Toronto Ontario Canada; ^3^ University of British Columbia Psychology Vancouver British Columbia Canada; ^4^ Division of Neurology Hospital for Sick Children Toronto Ontario Canada; ^5^ Holland Bloorview Kids Rehabilitation Hospital East York Ontario Canada

**Keywords:** autism, children, language, N400, semantic processing

## Abstract

**Introduction:**

The N400 is an electrophysiological component that reflects lexical access and integration of words with mental representations.

**Methods:**

Thirty‐five young children with a range of language capabilities (*n* = 21 neurotypical controls, 10 males, mean age = 6.3 ± 0.9 years; *n* = 14 children with autism, 12 males, mean age = 6.4 ± 1.1 years) completed an auditory semantic categorization paradigm to evoke the N400. Electroencephalograph (EEG) data were acquired with a 64‐channel electrode cap as children listened via ear inserts to binaurally presented single syllable words and decided whether the words were congruent (in) or incongruent (out) with a pre‐specified category. EEG data were filtered, epoched, and averaged referenced, and global field power (GFP) was computed. The amplitude of the N400 peak in the GFP was submitted to a multiple linear regression analysis.

**Results:**

N400 amplitude was found to predict language scores only for the children with ASD who have language scores in the normal range (*r* ^2^ = 0.72).

**Conclusions:**

This finding that N400 amplitude only predicted language scores in children with ASD and normal language scores suggests that these children may rely more on basic semantic processing (as reflected by the N400) and less on anticipating and predicting upcoming words. This suggests preferential utilization of a bottom‐up strategy to access higher order language.

## Graphical abstract

Young neurotypical and autistic children completed a listening task where they indicated if words belonged to a category or not, while an EEG was recorded from the scalp. A brain component called the N400 was found to predict language scores only in children with autism who showed normal language scores, suggesting they may rely on a bottom‐up word processing strategy as indexed by the N400. These findings have implications for language training strategies for children with autism spectrum disorder.

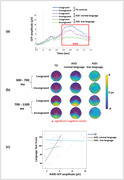

## 1 INTRODUCTION

Autism spectrum disorder (ASD) is an early onset neurodevelopmental disorder characterized by heterogeneous language capabilities that can range from relatively intact language (except for mild difficulties with language form, content or use) (Parsons et al., 2017) to minimally verbal (MV) to non‐verbal (NV) (Brignell et al., 2018; M. Dunn et al., 1999; Mody & Belliveau, 2013). While the Diagnostic and Statistical Manual of Mental Disorders, 5th Edition no longer includes language impairment as a diagnostic criterion for ASD, impairments in language are acknowledged as ubiquitous and subsumed under the other categories (American Psychiatric Association, 2013). The overall aim of the current study was to examine the neurobiology of semantic processing in children by using an auditory semantic categorization paradigm to evoke a component called the N400 with a specific objective to assess the relationship between this N400 component and language scores in young children across a spectrum of language capabilities.

Semantic processing involves encoding word meaning and comparisons to words in the same lexicon to create context for further processing. It has been reported that both semantic processing (Brignell et al., 2018; Mody & Belliveau, 2013; Tager‐Flusberg et al., 2005) and lexical access (Battaglia, 2012; Emmorey & Fromkin, 1988; Haebig et al., 2015; Taft, 2001) are impaired in ASD.

The neurobiology of semantic processing has been investigated using event‐related potentials (ERPs), which are time‐locked brain responses to cognitive processes (Sur & Sinha, 2009; Woodman, 2010). Specifically, a component called the N400, observed as a negative deflection at electrodes over central scalp regions, elicited ∼400 ms after stimulus onset, is thought to reflect the process of assessing incoming stimuli to determine if it is congruent with the preceding context (Kutas & Federmeier, 2011; Sur & Sinha, 2009). In adults, the amplitude of the N400 represents the additional resources required to access and integrate words with their context (Kutas & Federmeier, 2011; Kutas & Hillyard, 1980); therefore, incongruent words generate an N400 with a higher amplitude compared to congruent words. The difference in N400 amplitude between incongruent and congruent words is referred to as the “N400 effect” and is thought to be a reliable measure of semantic processing ability (Coderre et al., 2017; Kutas & Federmeier, 2011; Kutas & Hillyard, 1980). Further, the amplitude of the N400 effect is sensitive to degree of incongruency with more incongruent words generating a larger “N400 effect” (Benau et al., 2011).

The N400 component is observed in children; however, it is later, larger, of longer duration, and has a broader distribution (Atchley et al., 2006; Holcomb, 1993). This is due to the overlap of the N400 component with a second negative wave called the “late negative component” (LNC). The initial negativity (N400) is thought to index preliminary lexical access (Holcomb, 1993), and the subsequent component (LNC) is thought to represent integration of the word with mental representations (Silva‐Pereira et al., 2005). The amplitude of both of these components of the N400 is thought to represent bottom‐up semantic processing strategies that integrate words as they are presented, without any top‐down–driven prediction or anticipation processes (Kallioinen et al., 2016; Kutas & Federmeier, 2011; Mantegna et al., 2019).

Interestingly, the N400 effect is not reliably seen in children as it lacks sensitivity to semantic incongruencies (which require top‐down processing) although children can place words in their correct semantic category in behavioral tasks (Benau et al., 2011; Kallioinen et al., 2016). These studies suggest that the absence of the N400 effect does not indicate a deficiency in semantic processing in children. Nonetheless, studies investigating semantic processing in children with ASD have focused on the N400 effect. Not surprisingly, since the N400 effect is not well seen in typical development, it has been reported that children with ASD and high language capabilities also do not show an N400 effect (M. A. Dunn & Bates, 2005; McCleery et al., 2010; Ribeiro et al., 2013), and in children with ASD who are MV or NV, there are mixed reports of no N400 effect (Cantiani et al., 2016; DiStefano et al., 2019; Manfredi et al., 2020) or atypical N400 effects (primarily due to low amplitude and poor N400 components) (Cantiani et al., 2016; DiStefano et al., 2019; Manfredi et al., 2020). There have been no studies examining the N400 component, on its own, as this would be a better measure of semantic processing ability in children with ASD.

Further, ERP components that precede the N400 contribute to its generation. The obligatory cortical auditory evoked potentials, called the P1‐N1‐P2 complex, are a series of deflections in the ERP that occur before 300 ms and reflect cortical auditory processing (Munro et al., 2020; Ponton et al., 2000). Few studies have examined the P1‐N1‐P2 complex in children with ASD within the context of semantic processing. M. A. Dunn and Bates (2005) reported negligible differences in the amplitude and latency of the N1 component in high‐functioning children with ASD (M. A. Dunn & Bates, 2005). Cantiani et al. (2016) reported latency delays but no amplitude changes in the P1 component in minimally verbal/non‐verbal (MVL/NVL) children with ASD compared to typically developing (TD) children (Cantiani et al., 2016), suggesting intact afferent auditory input to the cortex. Thus, the contributions of early auditory responses to the subsequent generation of later responses related to spoken language were investigated in the present study.

In the current study, we used the N400 to examine the neurobiology of semantic processing in children with an auditory semantic categorization paradigm. Specifically, we assessed the relationship between the N400 and language scores in young children with varying language abilities. We also explored the contributions of the early cortical auditory evoked response peaks to the N400 component. The study's hypotheses were that (1) N400 amplitudes would decrease with decreasing language scores in children regardless of ASD diagnosis and that (2) early cortical responses, which represent auditory but not linguistic processing, would not be related to semantic processing as measured by language test scores.

## 2 METHODS

### 2.1 Participants

Forty‐three children ranging in age from 5 to 8 years were recruited, including 24 TD children and 19 children diagnosed with ASD. Child participants and their parents provided informed assent and consent, respectively. This study was approved by the Research Ethics Board at the Hospital for Sick Children (REB #1000061021).

TD participants were recruited by advertisements in the community, through social media and word of mouth. Exclusions included parent‐reported history of neurological, psychological, and/or psychiatric disorders; parent‐reported impairments in peripheral hearing or uncorrected vision; or academic, language, reading, or cognitive difficulties. None of the TD children reported taking medications.

Children with ASD were identified through the Province of Ontario Neurodevelopmental Network database at Holland Bloorview Kids Rehabilitation Hospital in Toronto. A diagnosis of ASD was made based on clinical observation and supported by scores on the Autism Diagnostic Observational Schedule—Generic/Autism Diagnostic Observational Schedule−2 (Lord et al., 2012) or the Autism Diagnostic Interview‐Revised (Lord et al., 1999). Exclusion criteria included parent‐reported impairments in peripheral hearing, uncorrected vision, and parent reported sensitivities that would preclude wearing the electroencephalograph (EEG) cap. The parents of both TD and ASD children were asked to confirm that they thought their child could sit still for three 5‐min intervals of EEG data acquisition. Medication use was not an exclusion, and children with ASD reported using Ritalin and Clonidine (*n* = 1), Arbaclofen (*n* = 1), Celexa (*n* = 1), and Montelukast (*n* = 1).

Two children with ASD did not complete the EEG protocol. EEG data for three TD and three ASD participants were excluded due to excessive movement or muscle artifact. The final sample consisted of 35 children including 21 TD children (10 males; mean age = 6.3 years ± 0.93; all right‐handed) and 14 children with ASD (12 males; mean age = 6.4 years ± 1.1; 11 right‐ and three left‐handed).

### 2.2 Language and cognitive assessments

On the day of EEG acquisition, receptive language was measured using the Peabody Picture Vocabulary Test, Fourth Edition (PPVT) (L. M. Dunn & Dunn, 2007) and cognitive functions assessed with the WPPSI‐IV (Wechsler, 2012). For four of the children with ASD, they were not able to complete the PPVT, and therefore their language scores were derived from other neuropsychological assessments including the PLS‐5 (*n* = 1) (Pearson, n.d.; Zimmerman et al., 2011), the WPPSI‐IV (verbal comprehension index; *n* = 2) (Wechsler, 2012), and the SB‐5 (verbal IQ; *n* = 1) (Roid, 2003). For the TD children, mean standardized language scores were 112 ± 11.1, while mean language scores were 95 ± 24.5 for the children with ASD, resulting in a significant difference in means between the TD and ASD children (p = 0.008). Six children with ASD were identified to have language impairments (Loucas et al., 2008; Snowling et al., 2020; Spaulding et al., 2006) with scores that fell more than one standard deviation below the standardized norm.

### 2.3 Stimuli for EEG task

Stimuli were single syllable words (44.1 kHz digitization rate, 16‐bit resolution, mono; 400–700 ms duration) spoken by a female native English speaker and delivered at 65 ± 3 dB SPL, binaurally through insert earphones. Words were derived from the MacArthur‐Bates Communicative Development Inventories: Words and Sentences Vocabulary checklist (CDI, n.d.) selected to be highly familiar to young children and fit within one of four semantic categories. Words and their corresponding categories are shown in Table 1.

**TABLE 1:** List of categories and stimulus words.

Animal	Food and drinks	Body parts	Action words (verbs)
Bear	Bread	Arm	Climb
Bee	Cake	Cheek	Draw
Bird	Corn	Chin	Drive
Cat	Egg	Ear	Jump
Cow	Grape	Foot	Kick
Dog	Gum	Head	Pull
Frog	Meat	Knee	Read
Hen	Milk	Leg	Run
Lamb	Pop	Mouth	Sing
Pig	Salt	Toe	Skate
Sheep	Soup	Tongue	Swim
Wolf	Toast	Tooth	Throw

Stimuli were presented using the Presentation software (Neurobehavioral Systems, Berkeley CA) in four blocks, corresponding to four pre‐determined categories. Each block consisted of 12 semantically congruent (i.e., in the correct category) and 12 incongruent words. In total, there were 96 trials consisting of 48 congruent and 48 incongruent stimuli. Words within each block were randomized at setup, but the same order was used for all participants. The order of blocks, however, was randomized for each participant. Stimuli were presented 2 s after the previous behavioral response. Participants could rest for as long as they needed between blocks.

### 2.4 Procedure

During the EEG recording, participants were seated 60 cm away from a computer screen where a visual reminder of the semantic category was displayed. Insert earphones were placed in the participants’ left and right ears, and participants were fitted with a high density 64‐channel Quick‐Cap Neo Net cap, with 10–20 configuration (Klem et al., 1999).

Participants completed an auditory semantic categorization task during EEG recording. At the beginning of each block, participants were verbally told the category by an examiner. This category was also displayed on the computer screen to reduce the need to hold this information in working memory. Participants listened to a list of words and indicated, as quickly as possible, their response with a button press on a computer mouse. Responses indicated whether the word was in‐category (semantically congruent), out‐of‐category (semantically in congruent), or unknown. As the participants were young children, they held the computer mouse with both hands and used their thumbs to press the button. Accuracy and reaction time (RT) were computed from these responses. For participants with lower cognitive or language abilities, who were unable to respond with a button press, the task was treated as a passive listening task, and the behavioral data for these participants were not included. A team member advanced the stimuli every 2–3 s if the child did not respond.

### 2.5 EEG acquisition and preprocessing

EEG data were recorded continuously using a NeuroScan v4.5 Synamps2 amplifier system (Compumedics, El Paso). The sampling rate was 1000 Hz and filtered from DC‐100 Hz. The recordings were referenced to an electrode placed between Cz and CPz electrodes for acquisition, and impedances for all electrodes were below 10 KΩ. Eye movements were recorded from four integrated bipolar leads for vertical and horizontal electrooculography. Trigger codes were sent from the stimulus computer via a parallel port to synchronize with the EEG data acquisition system. A team member sat next to the child throughout the EEG recording to ensure participant compliance and help minimize participant movement.

EEG data were imported and processed offline using the Fieldtrip toolbox in MATLAB (Oostenveld et al., 2011). Data were low pass filtered at 30 Hz and were referenced to an average of the mastoid electrodes (M1 + M2) for acquisition. Data were then epoched into individual trials relative to the onset of the word (−1.5 to 2 s) and downsampled to 500 Hz. Noisy channels were identified by visual inspection and replaced with interpolated proximal channels. An independent component analysis (ICA) algorithm was used to remove eye artifacts. Epochs with electrical activity greater than ±250 μV in any three or more channels were visually identified and rejected. The data for each participant were baseline corrected from 500 ms preceding the word to its onset at 0 ms and then averaged by condition.

### 2.6 Behavioral analyses

For the behavioral analyses, TD children and children with ASD (who were able to provide a response) were separated into two groups. Two separate 2 (cohort: TD vs. ASD) × 2 (condition: congruent vs. incongruent) analyses of variance (ANOVAs) were used to analyze behavioral data and evaluate differences in accuracy and RT. Effect size was calculated using partial eta squared.

### 2.7 EEG analyses

Global field power (GFP) is a reference‐independent measure of the strength of the neural responses across all electrodes and distributed over time (Skrandies, 1990) and was computed for each condition for each participant.

Cluster‐based permutations using paired *t*‐tests (*p* < .05, 1000 permutations) were computed using Fieldtrip functions (Maris & Oostenveld, 2007) to confirm the presence of an N400 component by identifying significant differences in electrical activity between baseline (−500 to 0 ms) and N400 active windows of interest (300–700 and 700–1100 ms) in the GFP. Cluster‐based permutations were also used to test for significant differences between conditions to identify an N400 effect in the two‐time windows of interest (300–700, 700–1100 ms).

ERP components of interest were automatically peak‐picked from the GFP within selected time windows (N400 component: 300–1100 ms; P1 of the P1‐N1‐P2 complex: 80–120 ms) for each of the 35 children and submitted to multiple linear regression analyses (nmle package in R). If no clear component was observed, the highest amplitude value was still picked from within the time window; therefore, values were obtained for every participant. A multiple linear regression tested whether the N400 GFP amplitude, age, or sex predicted language scores. A second multiple linear regression analysis tested whether the P1 GFP amplitude, age, or sex predicted language scores. Finally, a third linear regression tested whether the P1 GFP amplitude, age, or sex predicted the N400 GFP amplitude. Post hoc analyses were conducted using ANOVAs, and effect sizes were calculated using partial eta squared. Initial analyses were conducted with the full group, and exploratory regression analyses were conducted with the children divided into cohorts and cohort added as an additional predictor.

## 3 RESULTS

### 3.1 Demographic information

Demographic information for the final cohort is included in Table 2.

**TABLE 2:** Demographic information.

	TD Cohort	ASD Cohort
Sample size	21	14
Male:female	10:11	12:2
Age (mean ± SD) range	6.3 ± 0.93 5–8	6.4 ± 1.1 5–8
Handedness	21 right‐handed	11 Right‐handed, three left‐handed
Medication	None were taking medications	Ritalin and Clonidine (N = 1) Arbaclofen (N = 1) Valtrex, Tenox, Difluxan and Celexa (N = 1) Montelukast (N = 1)
Language assessment scores (mean ± SD)	112 ± 11.1	95 ± 24.5 (N = 12) High language: 114.5 ± 6.6 (N = 8) Low language: 67.8 ± 9.9 (N = 6)
Cognitive function scores (mean ± SD)	102 ± 11.9	91.4 ± 21

Abbreviations: ASD, autism spectrum disorder; TD, typically developing.

### 3.2 Behavioral results: Accuracy and reaction time

All children (*n* = 21) in the TD group completed the behavioral portion of the EEG task. Five of the 14 children in the ASD group were not able to complete the task; therefore, behavioural data for nine ASD participants were included.

For accuracy, there was a significant main effect of cohort (*F*[1,56] = 11.701, *p* = .001, *η* ^2^ = 0.17 with 90% CI = 0.05–0.32) with TD children (*M* = 86.5%, SD = 10.8) having higher accuracy than ASD children (*M* = 72.9%, SD = 19.5). There was no main effect of condition (*F*[1,56] = 0.240, *p* = .63, *η* ^2^ = 4.27e‐03 with 90% CI = 0.0–0.07), and the interaction effect was not significant (*F*[1, 56] = 0.238, *p* = .63, *η* ^2^ = 4.23e‐03 with 90% CI = 0.0–0.07).

For RT, there was a main effect of condition (*F*[1, 2785] = 9.613, *p* = .002, *η* ^2^ = 3.44e‐03 with 90% CI = 0.0–0.003) with the response to the congruent condition being faster (*M* = 1.8 s ± 0.007 SEM) than the incongruent (*M* = 1.9 ± 0.007); however, the effect size was negligibly small (*η* ^2^ = 3.44e‐03). There was no main effect of cohort (*F*[1,2785] = 3.556, *p* = .06, *η* ^2^ = 1.28e‐03 with 90% CI = 0.0–0.07) and no significant interaction (*F*[1,2785] = 0.030, *p* = .86, *η* ^2^ = 1.08e‐05 with 90% CI = 0.0–0.07).

### 3.3 EEG analyses

The EEG data from all 35 children (21 TD; 14 ASD) were used in the analyses. There was no significant difference between TD and ASD for the number of interpolated channels (TD: 1.05 ± 1 vs. ASD: 1.8 ± 2; *p* = .25), or number of trials in the congruent (TD: 46.5 ± 3 vs. ASD: 43.5 ± 6; *p* = .053) and incongruent (TD: 46.5 ± 3 vs. ASD: 43.6 ± 6; *p* = .07) conditions.

#### 3.3.1 N400 component

**FIGURE 1:**
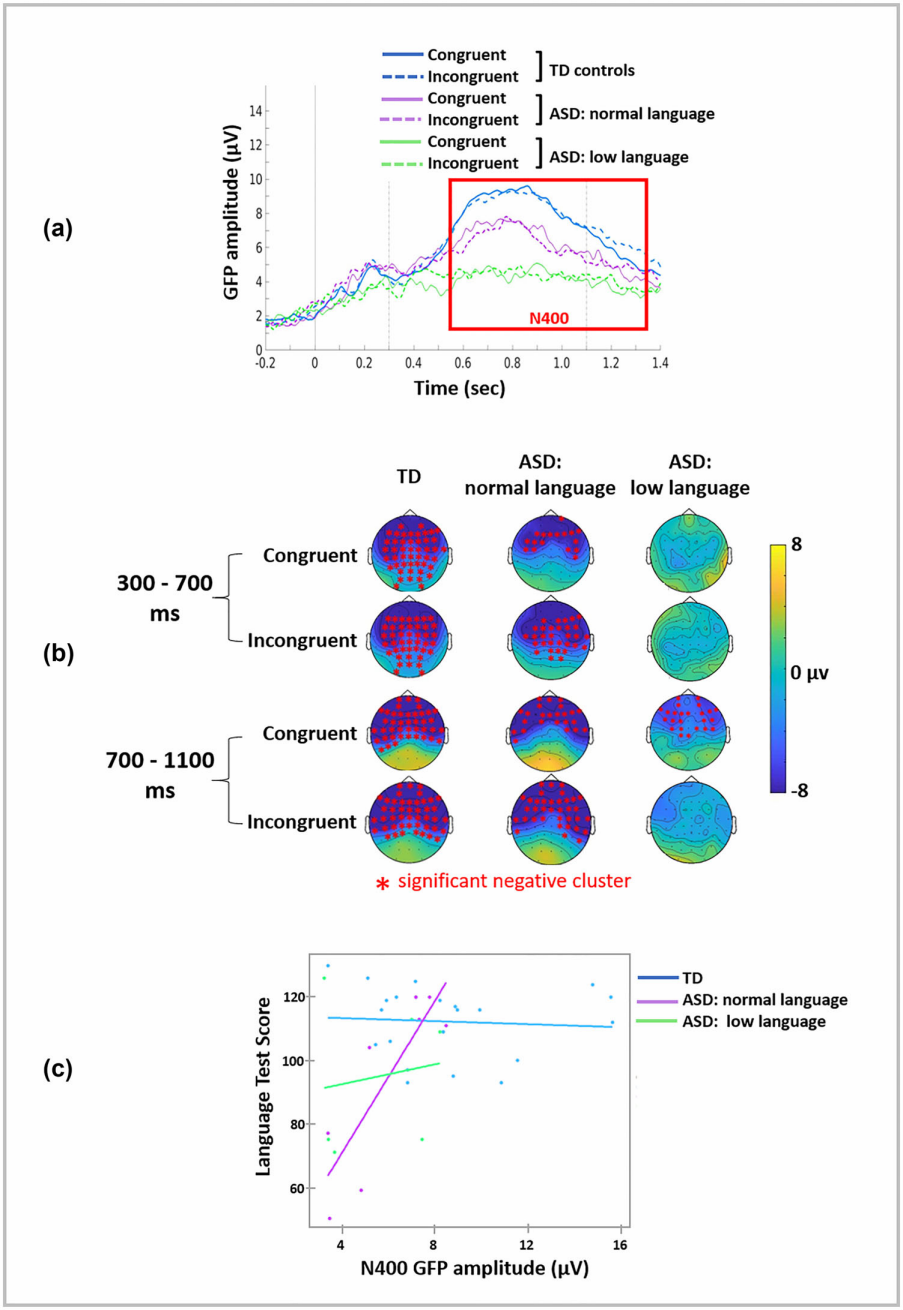
(a) Grand‐averaged global field plots (GFP) for the congruent (solid) and incongruent (dotted) conditions for the typically developing (TD) controls (blue), the autism spectrum disorder (ASD) group with normal language scores (purple), and the ASD group with low language scores (green). The N400 component is clearly seen in the first two groups. (b) Topographic plots (looking down on the head with the nose at the top) for the congruent and incongruent conditions for the TD control children, the children with ASD with normal language scores, and the children with ASD with low language scores in two time windows. Significant negative clusters from cluster‐based permutations are marked with red asterisks and show a broadly distributed (more fronto‐central) negativity in the first two groups, and a later, more frontal negativity in the low language group for the congruent condition. (c) Multiple regression analyses showed a significant N400 × cohort interaction. Correlation plots by cohort show that N400 amplitude is related to language test scores only for the ASD group with normal language scores.

The GFP was grand‐averaged by condition and cohort, plotted with topographic plots and presented in Figure 1. Examination of this figure shows that a clear N400 component was observed for children in the TD group (Figure 1a, blue line); however, there was great variability in the observation of this component in children with ASD with eight children having a detectable N400 and six children without a clearly identifiable N400 response. As an exploratory analysis, the ASD group was further subdivided into the children with language scores within the “normal range” (i.e., ± 1.5 SD; *n* = 8) and children with scores below 1.5 SD (*n* = 6). When divided this way, the grand‐averaged GFP is striking in that it showed a clear N400 in the TD and ASD with normal language groups and an absence of this ERP in the ASD with low language group (see Figure 1a). It is also clear from the tracings in Figure 1a that there was no difference in brain response between the congruent and incongruent conditions suggesting the absence of an N400 effect in all children. This was confirmed by cluster‐based permutations between the congruent/incongruent conditions which did not show any significant differences.

Figure 1b contains topographic plots. Cluster‐based permutations comparing baseline versus two active time windows (300–700 ms and 700–1100 ms) confirm the N400 component for the early (congruent: max sum = −178.97, *p* < .0001; incongruent: max sum = −149.24, *p* < .0001) and late (congruent: max sum = −253.05; *p* < .0001; incongruent: max sum: −292.52, *p* < .0001) time windows in the TD children as a broadly distributed negativity. In the ASD children with normal language scores, in both time windows, the early (congruent: max sum = −51.58, *p* = .02; incongruent: max sum = −96.35, *p* = .01) and late (congruent: max sum = −78. 59, *p* < .001; incongruent: max sum = −174.24, *p* < .001) components are observed as broadly distributed centro‐frontal negativities, while the children with ASD with low language scores only showed a negativity in the later time window (700–1100 ms) for the congruent condition (max sum = −92.50, *p* < .001) (see Figure 1b).

A multiple regression analysis that included all 35 children found that N400 amplitude (*p* = .15), age (*p* = .66) and sex (*p* = .23) were not significant predictors of the language score. In an exploratory analysis, where group was included as a predictor, the regression model of N400 amplitude on language scores was significant (F[7,27] = 3.81, *p* = .005) with a significant main effect for the N400 (*p* = .022) and the interaction of N400 × cohort (*p* = .0033). Age (*p* = .07) and sex (*p* = .51) were not significant predictors. The correlation plots by cohort are contained in Figure 1c and show that the regression is driven by the ASD group with normal language scores (*r* ^2^ = 0.72)(TD: *r* ^2^ = 0.005; low language: *r* ^2^ = 0.022).

#### 3.3.2 P1 component

**FIGURE 2:**
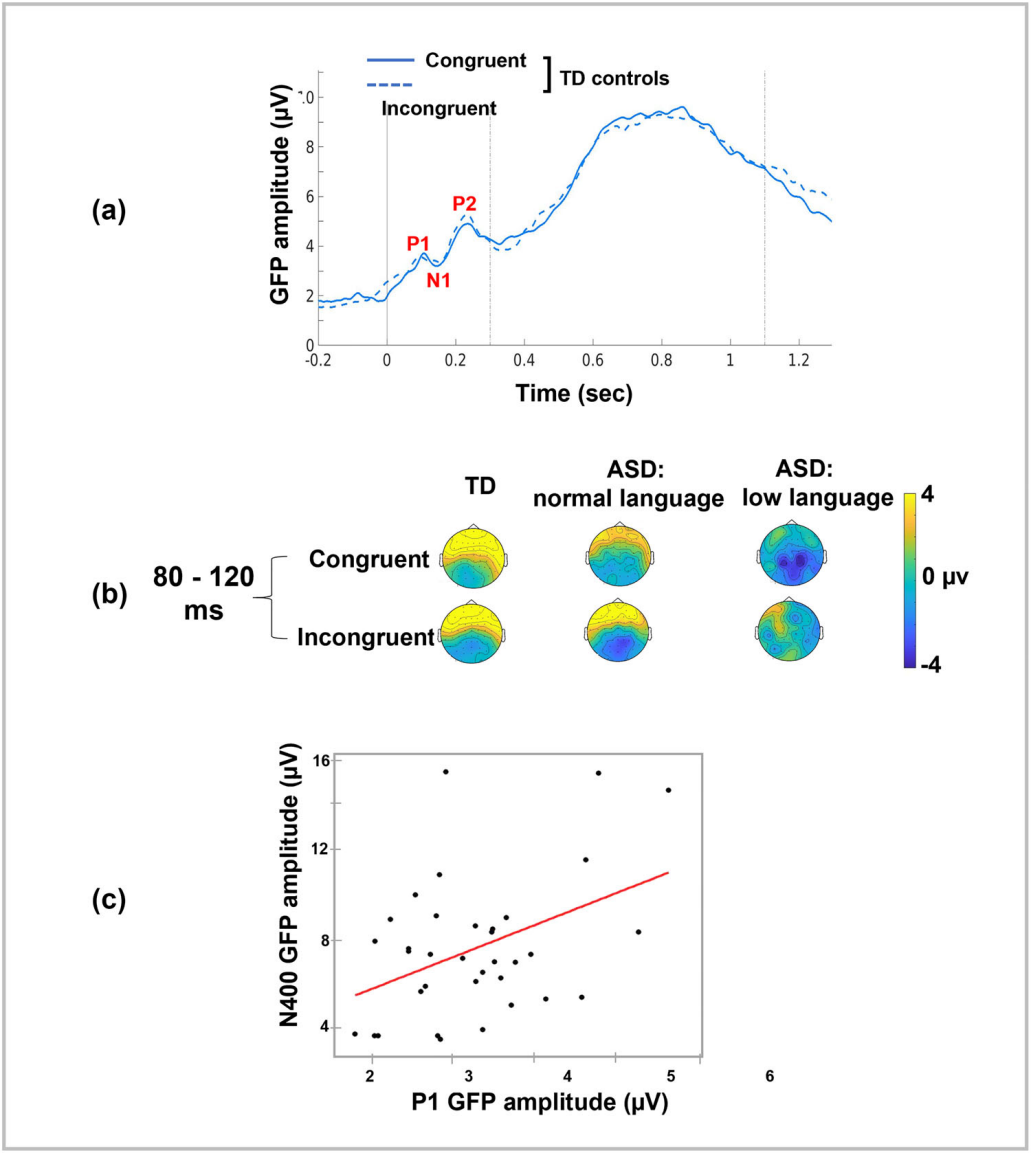
(a) Grand‐averaged global field plots (GFP) for the typically developing (TD) group for congruent (solid) and incongruent (dotted) conditions show clear early cortical potentials, namely the P1‐N1‐P2 complex. (b) Topographic plots (looking down on the head with the nose at the top) for the earliest peak, P1, for the two conditions and three groups suggest that differences occurred as early as the P1. (c) Multiple regression analysis found that P1 amplitude significantly predicted N400 amplitude, regardless of cohort.

In addition to the N400 component, inspection of the grand‐averaged GFP (Figure 1a) revealed divergence of the tracings between the groups in earlier time windows. Focusing on the TD group (Figure 2a), peaks corresponding to the P1 (80–120 ms), the N1b (130–180 ms), and P2 (200–260 ms) were observed. Topographic plots (Figure 2b) suggest a difference between groups as early as the P1; thus, subsequent analyses sought to explore the relationship between the P1 and the N400 component.

To examine the relationship described above, the P1 GFP amplitudes were submitted to a multiple regression analysis with P1 GFP amplitude, cohort, age and sex as predictors of the N400 component amplitude. The model was significant (F[7,27] = 2.623, *p* = .033; *R* ^2^ = 0.41). Post hoc testing showed that P1 amplitude significantly predicted N400 amplitude (*p* = .0083; Figure 2c). Cohort analysis showed a trend (*p* = .06; Figure 2d) toward significance, and sex (*p* = .12) and age (*p* = .47) were not significant. Multiple regression analyses with P1 GFP amplitude, age, and sex as predictors of language scores, with (*p* = .37) or without (*p* = .42) cohort included, were not significant.

## 4 DISCUSSION

In the current study, we used an auditory semantic categorization paradigm to evoke the N400 component in young children who had typical language scores and in children with ASD who had a range of language abilities. We also explored the P1 component of the early cortical auditory evoked response to determine its relationship to the N400 and to language scores.

As expected, all children who had language scores in the normal range (regardless of ASD status) demonstrated the presence of a sustained N400 component. This is consistent with evidence in the literature and most likely represents a combined N400 reflecting primary word access and retrieval with the LNC reflecting the integration of words with their mental representations (DiStefano et al., 2019; Silva‐Pereira et al., 2005). In addition, as expected, an N400 effect was not observed and is consistent with what is often seen in young children (Benau et al., 2011; Kallioinen et al., 2016).

We had hypothesized that N400 amplitude would be a significant predictor of language scores given the important relationship between semantic processing (Brignell et al., 2018; Mody & Belliveau, 2013; Tager‐Flusberg et al., 2005), specifically lexical access (Battaglia, 2012; Emmorey & Fromkin, 1988; Haebig et al., 2015; Taft, 2001), and language ability. However, this was not supported in our data. Instead, N400 amplitude only predicted language in children with ASD who had language scores within the normal range. One possibility is that, despite showing normal language scores, TD children and children with ASD use different mechanisms to process semantic information. Possibly, children with ASD rely heavily on basic semantic processing to access higher order language abilities, and therefore high amplitude N400 responses are related to higher language scores. It is known that language ability depends on a number of strategies incorporating both top‐down versus bottom‐up processes. One bottom‐up strategy involving integration of words as they appear without predicting and anticipating the upcoming words results in an all‐or‐none response (Kallioinen et al., 2016; Kutas & Federmeier, 2011; Mantegna et al., 2019). Using this bottom‐up strategy would generate a larger N400 and contribute to higher language scores. It may be that children with ASD rely heavily on bottom‐up processing, whereas TD children use a variety of strategies, and therefore N400 alone would not predict language scores. This interpretation needs to be directly tested using brain source imaging techniques and more complex language stimuli.

We had also hypothesized that early cortical auditory responses, namely the P1, would be related to language scores as intact cortical auditory processing is essential for language processing. Again, our hypothesis was not supported. Instead, we found that P1 amplitude predicted N400 amplitude, but not language scores, regardless of cohort. Considering the finding reported above where N400 amplitude predicted language in only the ASD cohort, it is not surprising that P1 amplitude did not predict language. However, the relationship between the P1 and N4 is interesting and suggests that children with poorer P1 responses generate poorer N4 responses. Traditionally, the P1 was thought to be a marker of cortical auditory maturation (Sharma et al., 2015). Recent research has observed that it is also sensitive to speech‐sound learning effects aiding predictive processes that are mediated in a top‐down fashion (Skoe et al., 2021). This research aligns with our data suggesting a reciprocal relationship between this early cortical response and the later higher order language response. Future studies should directly investigate this relationship.

While regression analyses using the full group of participants is interesting and the dissociation within the ASD group between the children who had language scores in the normal range compared to those with scores in the lower range emphasize the importance of exploring the full range of language abilities within ASD (Samson et al., 2015). This will capture a better representation of the observed heterogeneity. However, this approach runs the risk of floor effects as some of the children cannot do the task and/or have very low language scores.

In sum, the combined P1–N4 findings suggest that children with ASD who show more intact P1 responses are able to generate more intact N4 responses which allows them to use semantic processing to complete their basic language functions. Typically developing children also show this P1–N4 relationship; however, their language abilities arise from the culmination of other strategies and processes, not just semantic/bottom‐up processing. Finally, children with autism who cannot generate an intact P1 will struggle with language and present with minimal language function.

This is the first study to describe the relationship between the early cortical P1, the N4, and language scores as they present in a group of young children, with and without autism, and a wide range of language abilities. As a first study, there are a few limitations. Given the young age of our participants and the difficulty with recruiting such young children, we accepted all volunteers, especially those who were TD. For this reason, the groups are not matched with regard to sex and intelligence quotient. This is an issue that would need to be addressed in future studies. Further, the data need to be interpreted with caution due to the small sample size and exploratory analyses; however, these data address a gap in the literature regarding the N400 and ASD, and therefore provide a direction for future research. Future studies encompassing these groups, using larger sample sizes and methodologies that can examine the interplay of the underlying neurobiology with top‐down–mediated cognitive strategies are of utmost importance in allowing us to pinpoint the mechanism(s) responsible for language deficits and move toward the development of personalized targeted therapies for children with ASD, and children with language impairments.

## CONFLICT OF INTEREST STATEMENT

The authors declare no conflict of interest.

### PEER REVIEW

The peer review history for this article is available at https://publons.com/publon/10.1002/brb3.3158.

## Data Availability

The data that support the findings of this study are available on request from the corresponding author. The data are not publicly available due to privacy or ethical restrictions.

## References

[brb33158-bib-0001] American Psychiatric Association . (2013). Diagnostic and statistical manual of mental disorders (5th ed.). American Psychiatric Association. 10.1176/appi.books.9780890425596

[brb33158-bib-0002] Atchley, R. A. , Rice, M. L. , Betz, S. K. , Kwasny, K. M. , Sereno, J. A. , & Jongman, A. (2006). A comparison of semantic and syntactic event related potentials generated by children and adults. Brain and Language, 99(3), 236–246. 10.1016/j.bandl.2005.08.005 16226804

[brb33158-bib-0003] Battaglia, D. (2012). *Word association and semantic priming in individuals with autism spectrum disorders* [Ph.D. dissertation, City University of New York]. ProQuest Dissertations and Theses. https://search.proquest.com/docview/1114898101/abstract/86C46FD1FB614BF7PQ/1

[brb33158-bib-0004] Benau, E. M. , Morris, J. , & Couperus, J. W. (2011). Semantic processing in children and adults: Incongruity and the N400. Journal of Psycholinguistic Research, 40(3), 225–239. 10.1007/s10936-011-9167-1 21479571

[brb33158-bib-0005] Brignell, A. , Morgan, A. T. , Woolfenden, S. , Klopper, F. , May, T. , Sarkozy, V. , & Williams, K. (2018). A systematic review and meta‐analysis of the prognosis of language outcomes for individuals with autism spectrum disorder. Autism & Developmental Language Impairments, 3, 239694151876761. 10.1177/2396941518767610

[brb33158-bib-0006] Cantiani, C. , Choudhury, N. A. , Yu, Y. H. , Shafer, V. L. , Schwartz, R. G. , & Benasich, A. A. (2016). From sensory perception to lexical‐semantic processing: An ERP study in non‐verbal children with autism. PLoS One, 11(8), e0161637. 10.1371/journal.pone.0161637 27560378 PMC4999236

[brb33158-bib-0007] CDI . (n.d.). MacArthur‐Bates communicative development inventories . https://mb‐cdi.stanford.edu/

[brb33158-bib-0008] Coderre, E. L. , Chernenok, M. , Gordon, B. , & Ledoux, K. (2017). Linguistic and non‐linguistic semantic processing in individuals with autism spectrum disorders: An ERP study. Journal of Autism and Developmental Disorders, 47(3), 795–812. 10.1007/s10803-016-2985-0 28083778

[brb33158-bib-0009] DiStefano, C. , Senturk, D. , & Jeste, S. S. (2019). ERP evidence of semantic processing in children with ASD. Developmental Cognitive Neuroscience, 36, 100640. 10.1016/j.dcn.2019.100640 30974225 PMC6763343

[brb33158-bib-0010] Dunn, L. M. , & Dunn, D. M. (2007). Peabody picture vocabulary test (4th ed.) (PPVT‐4). APA PsycTests. 10.1037/t15144-000

[brb33158-bib-0011] Dunn, M. , Vaughan Jr , H. , Kreuzer, J. , & Kurtzberg, D. (1999). Electrophysiologic correlates of semantic classification in autistic and normal children. Developmental Neuropsychology, 16(1), 79–99. 10.1207/S15326942DN160105

[brb33158-bib-0012] Dunn, M. A. , & Bates, J. C. (2005). Developmental change in neutral processing of words by children with autism. Journal of Autism and Developmental Disorders, 35(3), 361–376. 10.1007/s10803-005-3304-3 16119477

[brb33158-bib-0013] Emmorey, K. D. , & Fromkin, V. A. (1988). The mental lexicon. In F. J. Newmeyer (Ed.), Linguistics: The Cambridge Survey: Volume 3: Language: Psychological and biological aspects (vol. 3, pp. 124–149). Cambridge University Press. 10.1017/CBO9780511621062.006

[brb33158-bib-0014] Haebig, E. , Kaushanskaya, M. , & Weismer, S. E. (2015). Lexical Processing in school‐age children with autism spectrum disorder and children with specific language impairment: The role of semantics. Journal of Autism and Developmental Disorders, 45(12), 4109–4123. 10.1007/s10803-015-2534-2 26210517 PMC4761424

[brb33158-bib-0015] Holcomb, P. J. (1993). Semantic priming and stimulus degradation: Implications for the role of the N400 in language processing. Psychophysiology, 30(1), 47–61. 10.1111/j.1469-8986.1993.tb03204.x 8416062

[brb33158-bib-0016] Kallioinen, P. , Olofsson, J. , Nakeva von Mentzer, C. , Lindgren, M. , Ors, M. , Sahlén, B. S. , Lyxell, B. , Engström, E. , & Uhlén, I. (2016). Semantic processing in deaf and hard‐of‐hearing children: Large N400 mismatch effects in brain responses, despite poor semantic ability. Frontiers in Psychology, 7, 1146. 10.3389/fpsyg.2016.01146 27559320 PMC4978721

[brb33158-bib-0017] Klem, G. H. , Lüders, H. O. , Jasper, H. H. , & Elger, C. (1999). The ten‐twenty electrode system of the International Federation. The International Federation of Clinical Neurophysiology. Electroencephalography and Clinical Neurophysiology. Supplement, 52, 3–6. 10590970

[brb33158-bib-0018] Kutas, M. , & Federmeier, K. D. (2011). Thirty years and counting: Finding meaning in the N400 component of the event related brain potential (ERP). Annual Review of Psychology, 62, 621–647. 10.1146/annurev.psych.093008.131123 PMC4052444 20809790

[brb33158-bib-0019] Kutas, M. , & Hillyard, S. A. (1980). Reading senseless sentences: Brain potentials reflect semantic incongruity. Science, 207(4427), 203–205. 10.1126/science.7350657 7350657

[brb33158-bib-0020] Lord, C. , Rutter, M. , DiLavore, P. C. , Risi, S. , Gotham, K. , & Bishop, S. (2012). (ADOS®‐2) Autism diagnostic observation schedule (2nd ed.). https://www.wpspublish.com/ados‐2‐autism‐diagnostic‐observation‐schedule‐second‐edition

[brb33158-bib-0021] Lord, C. , Rutter, M. , DiLavore, P. C. , & Risi, S. (1999). Autism diagnostic observation schedule—Generic. APA PsycTests. 10.1037/t17256-000

[brb33158-bib-0022] Loucas, T. , Charman, T. , Pickles, A. , Simonoff, E. , Chandler, S. , Meldrum, D. , & Baird, G. (2008). Autistic symptomatology and language ability in autism spectrum disorder and specific language impairment. Journal of Child Psychology and Psychiatry, and Allied Disciplines, 49(11), 1184–1192. 10.1111/j.1469-7610.2008.01951.x 19017030

[brb33158-bib-0023] Manfredi, M. , Cohn, N. , Sanchez Mello, P. , Fernandez, E. , & Boggio, P. S. (2020). Visual and Verbal narrative comprehension in children and adolescents with autism spectrum disorders: An ERP study. Journal of Autism and Developmental Disorders, 50(8), 2658–2672. 10.1007/s10803-020-04374-x 31974801

[brb33158-bib-0024] Mantegna, F. , Hintz, F. , Ostarek, M. , Alday, P. M. , & Huettig, F. (2019). Distinguishing integration and prediction accounts of ERP N400 modulations in language processing through experimental design. Neuropsychologia, 134, 107199. 10.1016/j.neuropsychologia.2019.107199 31545965

[brb33158-bib-0025] Maris, E. , & Oostenveld, R. (2007). Nonparametric statistical testing of EEG‐ and MEG‐data. Journal of Neuroscience Methods, 164(1), 177–190. 10.1016/j.jneumeth.2007.03.024 17517438

[brb33158-bib-0026] McCleery, J. P. , Ceponiene, R. , Burner, K. M. , Townsend, J. , Kinnear, M. , & Schreibman, L. (2010). Neural correlates of verbal and nonverbal semantic integration in children with autism spectrum disorders. Journal of Child Psychology and Psychiatry, and Allied Disciplines, 51(3), 277–286. 10.1111/j.1469-7610.2009.02157.x 20025622

[brb33158-bib-0027] Mody, M. , & Belliveau, J. W. (2013). Speech and language impairments in autism: Insights from behavior and neuroimaging. North American Journal of Medicine & Science, 5(3), 157–161. 24349628 10.7156/v5i3p157 PMC3862077

[brb33158-bib-0028] Munro, K. J. , Purdy, S. C. , Uus, K. , Visram, A. , Ward, R. , Bruce, I. A. , Marsden, A. , Stone, M. A. , & Van Dun, B. (2020). Recording obligatory cortical auditory evoked potentials in infants: Quantitative information on feasibility and parent acceptability. Ear and Hearing, 41(3), 630–639. 10.1097/AUD.0000000000000789 31633599 PMC7673631

[brb33158-bib-0029] Oostenveld, R. , Fries, P. , Maris, E. , & Schoffelen, J.‐M. (2011). FieldTrip: Open source software for advanced analysis of MEG, EEG, and invasive electrophysiological data. Computational Intelligence and Neuroscience, 2011, 1–9. 10.1155/2011/156869 21253357 PMC3021840

[brb33158-bib-0030] Parsons, L. , Cordier, R. , Munro, N. , Joosten, A. , & Speyer, R. (2017). A systematic review of pragmatic language interventions for children with autism spectrum disorder. PLoS One, 12(4), e0172242. 10.1371/journal.pone.0172242 28426832 PMC5398499

[brb33158-bib-0031] Pearson . (n.d.). PLS‐5 Preschool language scales (5th ed.). https://www.pearsonassessments.com/store/usassessments/en/Store/Professional‐Assessments/Speech‐%26‐Language/Preschool‐Language‐Scales‐%7C‐Fifth‐Edition/p/100000233.html

[brb33158-bib-0032] Ponton, C. W. , Eggermont, J. J. , Kwong, B. , & Don, M. (2000). Maturation of human central auditory system activity: Evidence from multi‐channel evoked potentials. Clinical Neurophysiology, 111(2), 220–236. 10.1016/S1388-2457(99)00236-9 10680557

[brb33158-bib-0033] Ribeiro, T. , Valasek, C. , Minati, L. , & Boggio, P. (2013). Altered semantic integration in autism beyond language. NeuroReport, 24(8), 414–418. 10.1097/WNR.0b013e328361315e 23629689

[brb33158-bib-0034] Roid, G. H. (2003). Stanford‐Binet intelligence scales. Riverside Publishing.

[brb33158-bib-0035] Samson, F. , Zeffiro, T. A. , Doyon, J. , Benali, H. , & Mottron, L. (2015). Speech acquisition predicts regions of enhanced cortical response to auditory stimulation in autism spectrum individuals. Journal of Psychiatric Research, 68, 285–292. 10.1016/j.jpsychires.2015.05.011 26037888

[brb33158-bib-0036] Sharma, A. , Glick, H. , Deeves, E. , & Duncan, E. (2015). The P1 biomarker for assessing cortical maturation in pediatric hearing loss: A review. Otorinolaringologia, 65(4), 103–114. 27688594 PMC5036577

[brb33158-bib-0037] Silva‐Pereira, J. , Rivera‐Gaxiola, M. , & Kuhl, P. K. (2005). An event‐related brain potential study of sentence comprehension in preschoolers: Semantic and morphosyntactic processing. Cognitive Brain Research, 23(2), 247–258. 10.1016/j.cogbrainres.2004.10.015 15820632

[brb33158-bib-0038] Skoe, E. , Krizman, J. , Spitzer, E. R. , & Kraus, N. (2021). Auditory cortical changes precede brainstem changes during rapid implicit learning: Evidence from human EEG. Frontiers in Neuroscience, 15, 718230. https://www.frontiersin.org/article/10.3389/fnins.2021.718230 34483831 10.3389/fnins.2021.718230 PMC8415395

[brb33158-bib-0039] Skrandies, W. (1990). Global field power and topographic similarity. Brain Topography, 3(1), 137–141. 10.1007/BF01128870 2094301

[brb33158-bib-0040] Snowling, M. J. , Hayiou‐Thomas, M. E. , Nash, H. M. , & Hulme, C. (2020). Dyslexia and developmental language disorder: Comorbid disorders with distinct effects on reading comprehension. Journal of Child Psychology and Psychiatry, 61(6), 672–680. 10.1111/jcpp.13140 31631348 PMC7317952

[brb33158-bib-0041] Spaulding, T. J. , Plante, E. , & Farinella, K. A. (2006). Eligibility criteria for language impairment. Language, Speech, and Hearing Services in Schools, 37(1), 61–72. 10.1044/0161-1461(2006/007) 16615750

[brb33158-bib-0042] Sur, S. , & Sinha, V. K. (2009). Event‐related potential: An overview. Industrial Psychiatry Journal, 18(1), 70–73. 10.4103/0972-6748.57865 21234168 PMC3016705

[brb33158-bib-0043] Taft, M. (2001). Lexical access, cognitive psychology of. In N. J. Smelser & P. B. Baltes (Eds.), International encyclopedia of the social & behavioral sciences (pp. 8743–8748). Pergamon. 10.1016/B0-08-043076-7/01538-2

[brb33158-bib-0044] Tager‐Flusberg, H. , Paul, R. , & Lord, C. (2005). Language and communication in autism. In Handbook of autism and pervasive developmental disorders: Diagnosis, development, neurobiology, and behavior (vol. 1, 3rd ed., pp. 335–364). John Wiley & Sons Inc.

[brb33158-bib-0045] Wechsler, D. (2012). WPPSI‐IV: Wechsler preschool and primary scale of intelligence (4th ed.). Pearson.

[brb33158-bib-0046] Woodman, G. F. (2010). A brief introduction to the use of event‐related potentials (ERPs) in studies of perception and attention. Attention, Perception & Psychophysics, 72(8), 2031–2046. 10.3758/APP.72.8.2031 PMC3816929 21097848

[brb33158-bib-0047] Zimmerman, I. L. , Steiner, V. G. , & Pond, R. E. (2011). PLS‐5 preschool language scales (5th ed.). Pearson/PsychCorp.

